# Impact of nutrient deficiency and harvesting strategy on biomass and phycocyanin production in *Spirulina* cultures

**DOI:** 10.3389/fbioe.2025.1546801

**Published:** 2025-03-24

**Authors:** Hooi Ren Lim, Kuan Shiong Khoo, Pau Loke Show

**Affiliations:** ^1^ Department of Chemical Engineering, Faculty of Engineering and Green Technology, Universiti Tunku Abdul Rahman, Kampar, Perak, Malaysia; ^2^ Department of Chemical and Environmental Engineering, Faculty of Science and Engineering, University of Nottingham Malaysia, Semenyih, Selangor Darul Ehsan, Malaysia; ^3^ Department of Chemical Engineering and Materials Science, Yuan Ze University, Taoyuan, Taiwan; ^4^ Department of Chemical Engineering, Khalifa University, Abu Dhabi, United Arab Emirates

**Keywords:** harvesting ratio, Spirulina, phycocyanin, scale-up, harvesting strategy

## Abstract

Recent research has focused on issues related to contamination, nutrient availability, and strain selection, but there has been insufficient focus on harvesting research. This study employed an integrated continuous cultivation and harvesting strategy for a *Spirulina* microalgae biorefinery. The effects of nutrient-deficiency, harvesting ratio, and NaNO_3_ addition on biomass concentration and productivity and phycocyanin accumulation of *Spirulina* were investigated. The lowest biomass productivity of 0.015 g/L/day was observed in *Spirulina* cultivated in NaNO_3_ deficient medium. A harvesting ratio of 10% showed a consistent range of harvested dry biomass weight (0.20–0.22 g). Addition of 2.50 g/L NaNO_3_ resulted in a significant increase in C-phycocyanin (C-PC) and allophycocyanin (APC) concentration from 34.37 mg/g to 68.35 and 27.08 to 33.23 mg/g, respectively. Biomass productivity of 1-L and 10-L batch culture was found to be 0.23 g/L/d and 0.21 g/L/d, respectively. Both 1-L and 10-L batch cultures showed a significant increase in phycocyanin accumulation due to the addition of 2.50 g/L of NaNO_3_. These findings highlight the feasibility of continuous cultivation and optimized harvesting for scalable biomass and phycocyanin production, offering valuable insights for industrial biorefineries that seek to enhance microalgae-based bioactive compound extraction.

## 1 Introduction

The global demand for the production of microalgae biomass as an alternative resource for applications such as biofuels, cosmetics, animal feed, carbon capture, pharmaceuticals, and food and nutritional supplements is currently expected to be at least 30,000 tons per year by 2030 (Amorim et al., 2021). *Spirulina* biomass accounts for 30% of the current 10,000 tons of total global algae biomass production (Lim et al., 2021). To realize this production target by 2030, researchers around the world have made many proposals to improve biomass productivity in the upstream processing of microalgae. Current industrial microalgae biomass production faces five challenges: contamination, cost, harvesting, nutrient availability, and strain selection (Venkata Subhash et al., 2022; Ummalyma et al., 2022).

Extensive research has explored alternative solutions to overcome issues of the selection, optimization, and formulation of cultivation medium in the microalgae biorefinery (Fábregas et al., 2000; Salunke et al., 2016). Dos Santos et al. (2016) reported that the addition of supplements (e.g., sugarcane vinasse) during microalgae cultivation increased microalgae biomass concentration by 23%, from 0.495 to 0.609 g/L, and yielded a higher protein content of 75%. Despite the selection and optimization of cultivation medium or addition of supplements to culture medium, the increase in biomass productivity is limited by different microalgae strains. Therefore, researchers have shifted their focuses by incorporating strategies involving genetic modification (Khoo et al., 2023). However, this is a relatively new field that requires much ongoing study and research before mutated species can be commercially utilized by industry (Fang et al., 2013). In addition, closed-system photobioreactors have reported that biomass productivity (12 g/m^2^/d) is higher than in open-pond systems (8 g/m^2^/d) due to the former’s closed environment and reduced contamination (Narala et al., 2016). However, there is a problem when scaling up the photobioreactor as biomass yield would decrease (Benner et al., 2022). Current research has focused on issues related to contamination, nutrient availability, and strain selection, but there has been insufficient research on harvesting.

Microalgae biomass harvesting methods such as coagulation and flocculation, centrifugation, electrical-based processes, and filtration have been studied (Singh and Patidar, 2018). Recent studies have focused extensively on optimizing these harvesting techniques to improve efficiency and reduce costs. However, researchers have also highlighted the advantages of continuous cultivation over batch and semi-batch strategies (Peter et al., 2022). In batch cultures, the cell composition may change over time as cells age and the bulk environment changes, resulting in harvested biomass of poor quality which may contain dead cells. However, continuous cultures enable efficient control of the growing environment, leading to tailor-made biomass composition at a constant and predetermined rate. Compared to batch mode (7.3 g/L/d), continuous cultivation systems offer higher productivity (42.6 g/L/d), 2.3 to 5 times higher, without accounting for the time required to clean and restart a batch culture (Fernandes et al., 2015; Gao et al., 2016). Despite the extensive research on harvesting methods and the benefits of continuous cultivation, there remains a critical gap in understanding the amount of biomass that should be harvested per unit time in a continuous system to optimize both the yield and sustainability of microalgae growth.

Therefore, our research here addresses the issue above by employing an integrated continuous cultivation and harvesting strategy for the biorefinery of *Spirulina* microalgae. This research aims to investigates the impact of different harvesting ratios (e.g., 10%, 20%, and 30%) on *Spirulina* cultivation. Additionally, it examines the effect of different concentrations of sodium nitrate (NaNO_3_) additions (e.g., 2.5 g/L, 3.5 g/L, and 4.5 g/L) on the phycocyanin (C-phycocyanin—CPC, and allo-phycocyanin—APC) accumulation in *Spirulina*. Phycocyanin has been studied for its various potential therapeutic properties, including antioxidant, anti-inflammatory, and immunomodulatory effects (ElFar et al., 2022). Based on findings, NaNO_3_ has been identified as the most important chemical for the growth of *Spirulina*. To improvise the harvesting strategy, instead of adding the entire fresh medium to replace the harvested biomass volume, we aimed to investigate whether the addition of just NaNO_3_ is sufficient to enhance the production of phycocyanin accumulation in the *Spirulina* microalgae, thereby reducing chemical usage in the system while optimizing phycocyanin production. Finally, the tested parameters of harvesting ratios and NaNO_3_ concentration were tested in different scales of 1-L and 10-L batch cultivation photobioreactor to evaluate and understand its effects on the biomass concentration, biomass productivity, harvested dry biomass, and phycocyanin accumulation. This research will provide insight on the impact of a continuous cultivation and harvesting strategy on biomass and phycocyanin production in a *Spirulina* microalgae biorefinery.

## 2 Materials and methods

### 2.1 Microalgae strains and growth conditions

*Spirulina platensis* microalgae were collected from Biolina Sdn. Bhd., Malaysia. Cultures were maintained in Zarrouk medium (Dineshkumar et al., 2016) with some modifications—NaHCO_3_: 18 g/L; NaNO_3_: 2.5 g/L; MgSO_4_.7H_2_O: 0.2 g/L; CaCl_2_.2H_2_O: 0.04 g/L; K_2_HPO_4_: 0.5 g/L; NaCl: 1 g/L; K_2_SO_4_: 1 g/L; FeSO_4_.7H_2_O: 0.01 g/L; EDTA: 0.08 g/L; micronutrient: 1 mL/L. The micronutrient consisted of H_3_BO_3_: 2.86 g/L; MnCl_2_.4H_2_O: 1.81 g/L; ZnSO_4_.7H_2_O: 0.222 g/L; Na_2_MoO_4_.2H_2_O: 0.3 g/L; CuSO_4_.5H_2_O: 0.07 g/L; Co(NO_3_)_2_.6H_2_O: 0.04 g/L.

*Spirulina* microalgae were inoculated and pre-cultured in 250-mL optimized Zarrouk medium for 7 days. The pre-cultured *Spirulina* microalgae were then transferred to a 1-L batch cultivation. The experiments were carried out in a 1-L photobioreactor. These 1-L batch cultivations were placed on a magnetic stirrer to ensure homogeneous mixing. The batch culture was cultivated under photoautotrophic growth conditions. The batch culture experiments were carried out at an illumination of approximately 3000 lux using LED lights. The light intensity was measured using a lux meter (UT383, UNI-T). Aeration was supplied to the batch cultivation via an air compressor (ACO-308, HAILEA, China). The air flow rate was maintained at 400 mL/min using an airflow regulator (Dwyer, Malaysia) throughout the experiment.

### 2.2 Scale-up to 10-L aerated batch cultures

A cylindrical closed-system photobioreactor with a 12 cm diameter and 150 cm height was fabricated by Donewell Resources Sdn. Bhd., Malaysia. The material used to fabricate the cultivation tank was acrylic plastic because of its transparency and chemical robustness (Huang et al., 2017). Transparency is crucial for *Spirulina* cultivation as it requires light for photosynthesis. Chemical robustness is important because of its chemical resistance to the many chemicals used to prepare the cultivation medium. The working volume of the cultivation tank was 10-L.

We prepared 1-L batch cultivation as the inoculum for 10-L batch cultivation. The batch culture was cultivated under photoautotrophic growth conditions. A nano-airstone (VN-132, Yek Fong Aquarium Accessories Sdn Bhd, Malaysia) was placed inside the 10-L cultivation tank to supply aeration via an air compressor for the *Spirulina* culture. The air flow rate was maintained at 20 L/min using an airflow regulator throughout the experiment. The 10-L batch culture experiments were carried out with an illumination of approximately 2000 lux with four LED light tubes installed around the cultivation tank. The intensity of light was measured with a lux meter. All scale-up cultures were supplemented with Zarrouk media.

### 2.3 Harvesting ratio strategy

#### 2.3.1 Effects of Zarrouk medium

Conventional methods typically harvest the entire batch at once. However, this study explored a gradual harvesting approach, starting with 10% of the biomass and progressively increasing the harvesting ratio (10%, 20%. 30%) until the batch culture could no longer maintain a consistent daily biomass production for three consecutive days. Each cultivation batch was cultured for 10 days before consecutive harvesting. The batch cultivation was replenished with a quantity of fresh culture medium equal to that harvested. For each batch cultivation, the absorbance of the culture was analyzed daily. The harvested fresh culture was centrifuged to remove the supernatant, and the resulting biomass was freeze-dried. After the optimum harvesting ratio was determined, it was tested in a large-scale 10-L *Spirulina* cultivation tank.

#### 2.3.2 Effects of sodium nitrate addition

Instead of adding the culture medium, only NaNO_3_ was added since it is the primary source of nitrate in Zarrouk medium with a standard concentration of 2.5 g/L. Previous research has demonstrated increased biomass growth, phycocyanin, and allo-phycocyanin accumulation in nitrate fed-batch phototrophic cultivation of *A. platensis* FACHB-314 (Manirafasha et al., 2018). Therefore, it is reasonable to explore higher concentrations of 2.5–4.5 g/L to assess its impact on biomass production and phycocyanin accumulation.

The harvesting ratio was fixed at 10% based on previous optimized harvesting ratios conducted in 1-L *Spirulina* cultivation. The effect of concentration of NaNO_3_ (2.5, 3.5, and 4.5 g/L) on biomass concentration and phycocyanin accumulation were studied. Each batch of cultivation was cultured for 10 days before harvesting. The batch cultivation was replenished with fresh NaNO_3_ solution after harvesting. After the optimum NaNO_3_ addition was determined, it was tested in a large 10-L Spirulina cultivation tank. Table 1 shows an overview of the operating parameters investigated for the production of phycocyanin from *Spirulina* microalgae.

**TABLE 1:** Operating parameters of *Spirulina* cultivation for producing phycocyanin.

No.	Operating parameter	Variables
1	Effect of nutrient-deficient media	NaHCO_3_, NaNO_3_, NaCl, K_2_SO_4_, K_2_HPO_4_, MgSO_4_.7H_2_O, FeSO_4_.7H_2_O and EDTA, CaCl_2_.2H_2_O, micronutrient
2	Harvesting ratio (1-L)	10%, 20%, 30%
3	Effect of NaNO_3_ addition (1-L)	2.5 g/L, 3.5 g/L, 4.5 g/L
4	Scale-up 10-L photobioreactor	Comparison of 1-L and 10-L + Harvesting ratio (10%) + Effect of NaNO_3_ addition (2.5 g/L)

### 2.4 Biomass sampling

The cultivation progress was monitored by collecting biomass samples. The absorbance of the biomass concentration of the culture was measured with a UV–Vis spectrophotometer (UV-1800, Shimadzu, Japan) at a 688 nm wavelength (Chen et al., 2016). However, the UV–Vis spectrophotometer has a limitation of reading absorbance values up to 4, so the samples had to be diluted ten times and the average taken to ensure accurate readings. Fresh samples were used to determine the biomass concentration and productivity. Samples for phycocyanin extraction were stored at −20 ℃ until further analysis.

### 2.5 Growth and biomass productivity determination

#### 2.5.1 Biomass determination with standard calibration curve

A sample of mature *Spirulina* medium was subjected to dilutions of 20%, 40%, 60%, 80%, and 100% with deionized water in 15-mL centrifuge tubes. The absorbance of each diluted sample was measured and recorded using a UV-Vis spectrophotometer at a specific wavelength of 688 nm. Subsequently, the samples were centrifuged to remove the supernatant and rinsed with deionized water to remove excess salt, and the resulting biomass was freeze-dried. The dry biomass weight was then measured and recorded. A standard calibration graph was generated by plotting the dry biomass weight against absorbance. The equation obtained from this graph was used to convert all measured absorbance data into dry biomass weight (g/L). The standard calibration graph allows us to estimate the dry biomass weight of *Spirulina* from the absorbance data. Finally, the remaining cultivated *Spirulina* biomass was poured into 50-mL centrifuge tubes and centrifuged at 6,500 rpm for 5 min (Koyande et al., 2019). The supernatant was then discarded, and the wet biomass was freeze-dried and stored at −20 ℃ for further analysis. The freeze-dried biomass was used for proximate analysis to determine the phycocyanin content.

#### 2.5.2 Biomass productivity and specific growth rate

The biomass productivity (P_b_) and specific growth rate (µ) were calculated based on Equations 1 and 2 (Chew et al., 2018) as follows :

$$P_{b}\;\left(\text{mg}/L/\text{day}\right)=\left(N_{2}-N_{1}\right)/\left(t_{2}-t_{1}\right),$$ (1)

$$µ\;\left(d^{-1}\right)=\ln\left(N_{2}/N_{1}\right)/\left(t_{2}-t_{1}\right),$$ (2)

where N_1_ and N_2_ are biomass concentration (g/L) at time t_1_ and t_2_, respectively.

#### 2.5.3 Phycocyanin quantification

Two types of phycocyanin were analyzed: C-phycocyanin (CPC) and allo-phycocyanin (APC). Phosphate buffer was added to a measured quantity of freeze-dried *Spirulina* biomass in a 0.009% (m/v) biomass-to-solvent ratio. The use of phosphate buffer as the solvent was chosen in this study as it has been demonstrated to improve the structural stability of protein molecules during the extraction process, which may be subject to various forces that could lead to degradation or denaturation (G-Bioscience, 2019). The mixture was homogenized using a vortex mixer and then subjected to ultrasonic sonication at a frequency of 35 kHz for 5 min (Chew et al., 2019). This process disrupted the cell walls of the *Spirulina* cells and released the phycocyanin composition. The sonicated sample was then centrifuged at 6,500 rpm for 5 min, and the supernatant was collected for further analysis. The CPC and APC concentrations in the supernatant were quantified using a UV–Vis spectrophotometer at various wavelengths. Food grade phycocyanin was used as a standard to validate Equations 1 and 2 for quantifying phycocyanin concentrations.

CPC and APC concentrations were calculated based on Equations 3 and 4 (Bennett and Bogorad, 1973).

$$CPC\;\left(mg/ml\right)=\frac{{OD}_{615}-0.474\times {OD}_{652}}{5.34},$$ (3)

$$APC\;\left(mg/ml\right)=\frac{{OD}_{652}-0.208\times {OD}_{615},}{5.09}$$ (4)

where OD_615_ and OD_652_ are the optical density wavelengths at 615 nm and 652 nm, respectively.

The purities of CPC and APC were calculated based on Equations 5 and 6 (Abalde et al., 1998).

$${Purity}_{CPC}=\frac{{OD}_{620}}{{OD}_{280},}$$ (5)

$${Purity}_{APC}=\frac{{OD}_{652}}{{OD}_{280},}$$ (6)

where OD_280_, OD_620_, and OD_652_ are the optical density wavelengths at 280 nm, 625 nm, and 652 nm, respectively.

### 2.6 Statistical analysis

All cultures were performed in duplicate and average values were reported. The values were then expressed in terms of mean and standard deviation. Subsequently, the data were subjected to one-way analysis of variance (ANOVA) using Microsoft Excel software to assess any significant differences between the groups with a p-value of less than or equal to 0.05.

## 3 Results and discussion

### 3.1 Effects of nutrient-deficient media on 1-L *Spirulina* cultivation

**FIGURE 1:**
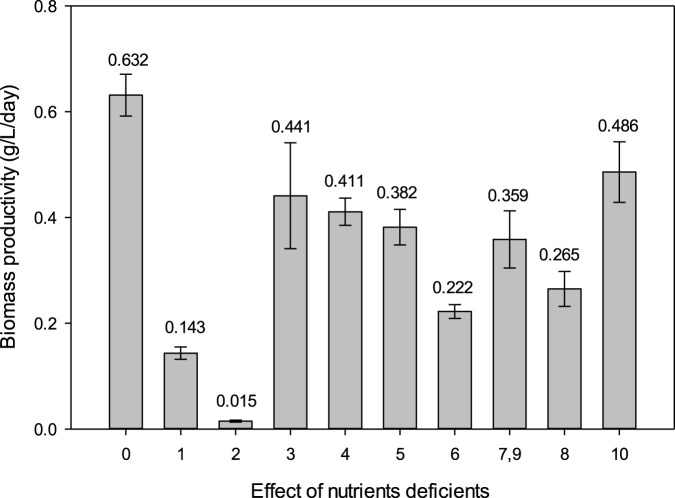
Effect of nutrient-deficient media on biomass productivity of *Spirulina* cultivation. [0] Zarrouk medium as control, [1] NaHCO_3_, [2] NaNO_3_, [3] NaCl, [4] K_2_SO_4_, [5] K_2_HPO_4_, [6] MgSO_4_.7H_2_O, [7, 9], FeSO_4_.7H_2_O and EDTA, [8] CaCl_2_.2H_2_O, and [10] micronutrient.

*Spirulina* cultivation in NaNO_3_-deficient medium has the lowest biomass productivity of 0.015 ± 0.002 g/L/day (Figure 1. The *Spirulina* cultivation in NaNO_3_-deficient medium did not survive the 10 days of cultivation. This is because NaNO_3_ is an essential nutrient for *Spirulina* cultivation as it is the nitrogen source in the cultivation medium. Nitrogen is important for the synthesis of proteins, nucleic acids, and chlorophyll content in microalgae (Wu and Miao, 2014). Nitrogen limitation will result in decreased photosynthesis, protein synthesis, and increase in lipid and carbohydrate synthesis (Li et al., 2012). Therefore, NaNO_3_-deficient medium shows the lowest biomass productivity. Moreover, phycocyanin is a type of pigment–protein complex. Since NaNO_3_ is responsible for protein synthesis, this study also investigated the effect of NaNO_3_ concentration on phycocyanin accumulation in *Spirulina*.

NaHCO_3_-deficient medium also exhibits a low biomass productivity of 0.143 ± 0.012 g/L/day. The *Spirulina* cultivation in NaHCO_3_-deficient medium survived the 10 days of cultivation. NaHCO_3_ is an essential nutrient for *Spirulina* cultivation because it contributes carbon to the cultivation medium. Only *Spirulina* is able to utilize carbonate or bicarbonate, supplied in the form of salts (Becker, 1994). The mechanism of NaHCO_3_ utilization as an inorganic carbon source for microalgae differs from CO_2_ uptake. The bicarbonate transporters are embedded in the chloroplast envelope and plasma membrane in microalgal cells. Bicarbonate ions (HCO^3−^) are converted to CO_2_ by carbonic anhydrase in the periplasmic space, which is then absorbed and utilized by microalgal cells (Mondal et al., 2017). Moreover, when NaHCO_3_ dissociates in the water, it produces hydroxyl ions in the medium that help sustain a high pH 9-10 throughout the cultivation condition (Michael et al., 2019). Research has reported that *Chlorella vulgaris*, under nutrient-sufficient condition with NaHCO_3_ as the carbon source, was able to reach 1.92 g/L maximum cell density—almost twice the biomass (1.04 g/L) cultivated under aeration alone (Abedini Najafabadi et al., 2015). *Spirulina* was able to grow in NaHCO_3_-deficient medium but resulted in low biomass productivity. NaHCO_3_ exhibits a dual role which acts as a carbon source and maintains a high pH condition suitable for *Spirulina* cultivation.

Beyond NaNO_3_ and NaHCO_3_, other nutrients such as K_2_HPO_4_ also play an important role in *Spirulina* growth. K_2_HPO_4_ serves as a phosphorus source, which is essential for cellular metabolism such as energy conversion, photosynthesis, and signal transduction (Nyabuto et al., 2015). Previous studies have shown that optimizing phosphate concentrations have significant effects on chlorophyll, metabolite, and carotenoid content (Abd El-Monem et al., 2021). Moreover, phosphorus deficiency has been reported to reduce photosynthetic efficiency and protein synthesis due to irregular nucleic acid synthesis, while shifting the cell’s focus to the synthesis of storage products, including carbohydrates and hydrocarbons (Dorry et al., 2024). Therefore, the balance of nutrients such as NaNO_3_, NaHCO_3_, and K_2_HPO_4_ is crucial not only for biomass productivity but also for the synthesis and purity of high-value compounds such as phycocyanin.

Overall, the deficiency of each nutrient has been shown to have lower biomass productivity than a control (0.632 ± 0.039 g/L/day) that contains the complete Zarrouk medium. NaNO_3_ and NaHCO_3_ have been identified as the most important nutrient sources for *Spirulina* cultivation. Although Zarrouk has been an established medium for cultivating cyanobacteria since 1966, not much information has been released regarding the formulation of Zarrouk medium. Therefore, the current finding is important in understanding the nutrients of Zarrouk medium on the biomass productivity of *Spirulina*. Further research would optimize the concentration of NaNO_3_, NaHCO_3_, and other nutrients to achieve a cost-effective cultivation medium for *Spirulina*. Statistical ANOVA shows that nutrient deficiency has a significant effect (p < 0.05) on the biomass productivity of *Spirulina*.

### 3.2 Effects of harvesting ratio on 1-L *Spirulina* cultivation

**FIGURE 2:**
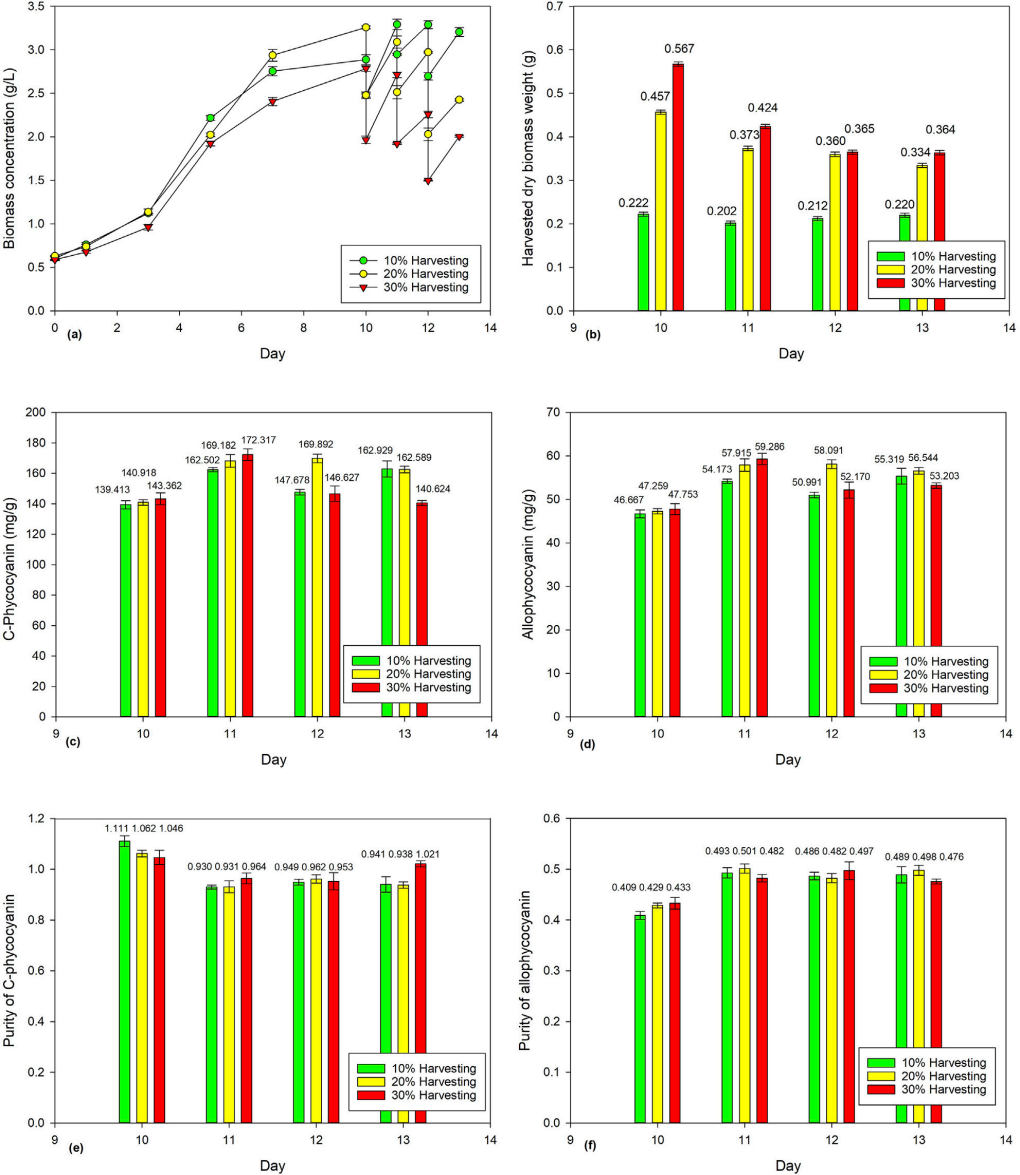
Effect of harvesting ratio (10%, 20%, and 30%) on *Spirulina* cultivation: **(a)** biomass concentration, **(b)** harvested dry *Spirulina* biomass weight, **(c)** C-phycocyanin concentration, **(d)** allo-phycocyanin concentration, **(e)** C-phycocyanin purity, and **(f)** allo-phycocyanin purity.

Figure 2a shows the biomass concentration of *Spirulina* cultivated for 10 days before being subjected to different harvesting ratios (e.g., 10%, 20%, and 30%). Figure 2b shows the dry biomass weights of *Spirulina* harvested from Days 10–13 consecutively at different harvesting ratios (10%, 20%, and 30%). Only the 10% harvesting ratio shows a more consistent harvested dry biomass weight, ranging from 0.2016 g to 0.2222 g. With the 20% and 30% harvesting ratios, the harvested dry biomass decreases from 0.4566 g to 0.3344 g and 0.5671 g to 0.3635 g, respectively. A lower harvesting ratio resulted in longer hydraulic retention time in which higher biomass density would be accumulated; thus, more nutrients would be assimilated, and the biomass concentration would be able to recover in a day. However, when the harvesting ratio increases, lesser biomass density is accumulated, and thus lesser nutrients would be assimilated, resulting in decreases of harvested dry biomass weights over the four consecutive days. Min et al. (2011) reported that 25% and 33% harvesting ratios showed better nutrient removal than half of its harvesting rate.

The current study investigated the harvesting ratio, but further research may investigate the harvesting frequency, such as harvesting intervals of 1 , 3 , and 5 days on *Spirulina* cultivation. Cai et al. (2013) reported that the biomass productivity of *Nannochloropsis salina* increased from 87.4 to 132.1 mg/L/d when the harvesting interval reduced from 3° to 1 day. This is because the long harvesting interval used to increase the number of larger clumps would raise the self-shading of cells and interfere with light absorption efficiency and the photosynthetic efficiency of the microalgae due to the reduced biomass productivity (Ishika et al., 2021). To conclude, a low harvesting ratio and frequent harvesting would be a good harvesting strategy to produce a high amount of biomass.

The respective reported CPC and APC concentrations ranged from 139.413 to 172.317 mg/g and 46.667 to 59.286 mg/g (Figures 2c, 2d). The reported CPC and APC purities ranged from 0.930 to 1.111 and 0.409 to 0.501, respectively. Statistical ANOVA analysis showed no significant effect (p > 0.05) on phycocyanin concentration or purity, while the effect of harvesting ratio has a significant effect (p < 0.05) on the harvested dry biomass weight of *Spirulina*. Overall, the findings provided valuable insights on the impact of harvesting ratio on harvested dry biomass and phycocyanin accumulation in *Spirulina*.

### 3.3 Effects of NaNO_3_ addition on phycocyanin concentration in 1-L *Spirulina* cultivation

**FIGURE 3:**
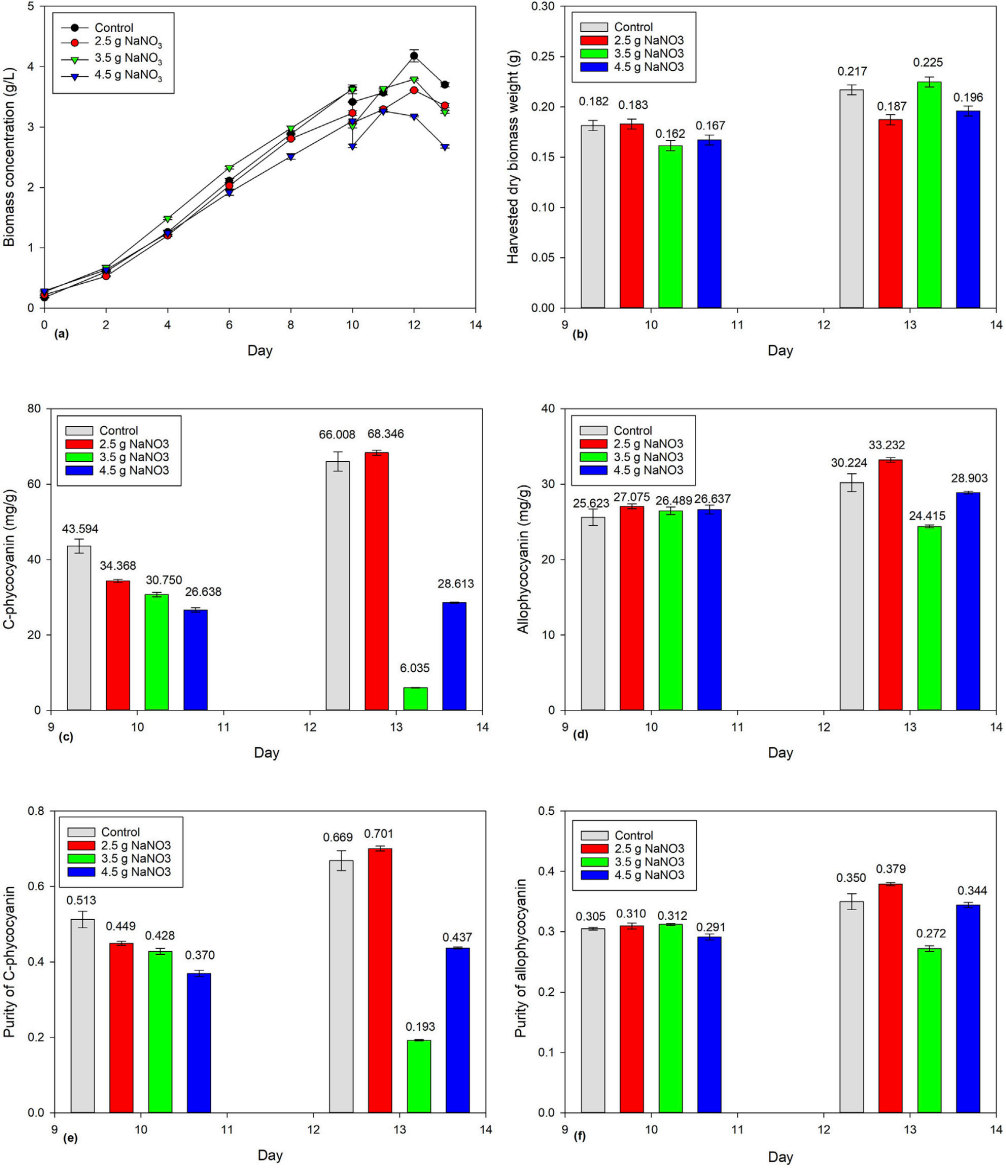
Effect of NaNO_3_ addition (2.5 g/L, 3.5 g/L, and 4.5 g/L) on *Spirulina* cultivation: **(a)** biomass concentration, **(b)** harvested dry *Spirulina* biomass weight, **(c)** C-phycocyanin concentration, **(d)** allo-phycocyanin concentration **(e)** C-phycocyanin purity, and **(f)** allo-phycocyanin purity.

Figure 3a shows the biomass concentration of *Spirulina* cultivated for 10 days before being subject to a harvesting ratio of 10%. An equal volume of NaNO_3_ (2.5 g/L, 3.5 g/L, and 4.5 g/L) was added back and cultivated for another 3 days to study its effect on biomass concentration, harvested dry biomass weight, and phycocyanin accumulation. After adding NaNO_3_, all four-batch cultivations showed a similar trend, with the biomass concentration continuing to increase from Days 10 to 12 but decreasing on Day 13. Therefore, the batch cultivations were stopped and harvested. Figure 3b shows the harvested dry biomass weight increasing slightly. On the other hand, the addition of 2.5 g/L of NaNO_3_ showed a significant increase in C-PC and APC concentration from 34.368 to 68.346 mg/g and 27.075 to 33.232 mg/g, respectively (Figure 3c). The control also showed significant increase in C-PC and APC concentration from 43.594 to 66.008 mg/g and 25.623 to 30.224 mg/g, respectively. However, the addition of 3.5 g/L of NaNO_3_ showed a significant decrease in C-PC concentration from 30.750 to 6.035 mg/g. Moreover, the addition of 4.5 g/L NaNO_3_ showed a slight increase in C-PC and APC concentration from 26.638 to 28.613 mg/g and 26.637 to 28.903 mg/g, respectively.

The current finding shows that both control and the addition of 2.5 g/L of NaNO_3_ increased phycocyanin accumulation. The control used Zarrouk medium to replace the 10% harvested *Spirulina* biomass. Instead of using the Zarrouk medium consisting of many chemicals, the current study shows that 2.5 g/L of NaNO_3_ only is capable of increasing phycocyanin accumulation. This is because phycocyanin is a type of pigment–protein complex, and NaNO_3_ is a nitrogen source that is responsible for the synthesis of proteins, nucleic acids, and chlorophyll in microalgae (Wu and Miao, 2014). Mousavi et al. (2022) found that medium supplemented with ammonium nitrogen sources have a slightly better growth rate than nitrogen sources (i.e., NaNO_3_), suggesting that ammonium plays a key role in supplying cells with the nitrogen source.

However, the concentration of 3.5 g/L NaNO_3_ onward showed no significant increase in phycocyanin accumulation. Sakawduan et al. (2019) reported a similar trend, with CPC concentration increasing from 25.5 to 44.59 mg/L/d when NaNO_3_ concentration increases from 1.5 to 3.5 g/L. Conversely, the CPC concentration decreases to 8.79 mg/L/d at 4.5 g/L of NaNO_3_. Vieira Costa et al. (2001) reported that when NaNO_3_ increases from 0.85 g/L to 2.55 g/L, the final biomass concentration increases from 1.559 g/L to 1.992 g/L, but the final biomass concentration decreases to 1.628 g/L when the NaNO_3_ is 4.25 g/L. It can be concluded that the tolerance level of NaNO_3_ concentration is approximately 3.5 g/L. Manirafasha et al. (2018) suggested that the nitrate concentration in culture should be kept between 1.2 and 1.6 g/L for high phycocyanin accumulation. It also worth noting that Sakawduan et al. (2019) and Mousavi et al. (2022) studied the effect of nitrogen sources during the active cultivation cycle whereas the current study investigated the effect of NaNO_3_ on the post-cultivation cycle. Further research is needed to explore alternative nitrogen sources (i.e., urea, ammonium nitrate, and ammonium sulfate) on the post-cultivation cycle and to optimize the conditions to achieve the maximum phycocyanin accumulation in *Spirulina* cultures.

The current finding has demonstrated that the addition of 2.5 g/L of NaNO_3_ after post-cultivation has the ability to enhance phycocyanin accumulation in *Spirulina* microalgae. This strategy offers a cost-effective solution for the microalgae industry by reducing the use of chemicals in achieving a continuous cultivation process. Statistical ANOVA analysis shows that there was no significant effect (p > 0.05) on phycocyanin purity, while the effect of harvesting ratio has significant effect (p < 0.05) on the harvested dry biomass weight of *Spirulina*.

### 3.4 Comparison of 1-L and 10-L *Spirulina* cultivation volume

#### 3.4.1 Biomass productivity comparison

**FIGURE 4:**
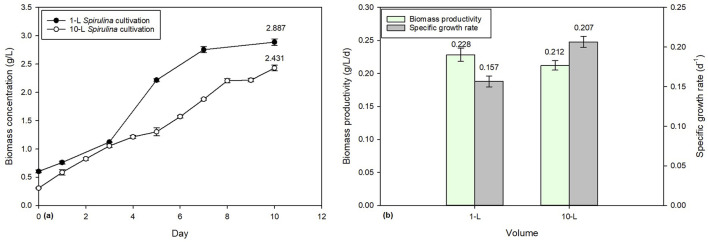
Comparison of 1-L and 10-L *Spirulina* cultivation: **(a)** biomass concentration and **(b)** biomass productivity.

Over the course of a 10-day cultivation period, the biomass concentration of *Spirulina* in 1-L and 10-L batch cultivations was determined to be 2.887 g/L and 2.431 g/L, respectively (Figure 4a. The biomass productivity of 1-L and 10-L batch cultivations was found to be 0.2284 g/L/d and 0.2122 g/L/d, respectively (Figure 4b). These findings suggest that the smaller scale 1-L system is more productive than the larger 10-L system, despite the latter having a larger volume for cultivation. When cultivating microalgae on a large scale, challenges arise, such as ensuring equal light distribution and mixing, providing sufficient gas exchange, and addressing the accumulation of soluble algal products in the culture medium (Jerney and Spilling, 2020).

To address the scaling challenges in larger scale systems, several engineering solutions can be implemented to enhance biomass productivity. First, to ensure more even light distribution across the culture, the use of light-emitting diodes (LEDs) with optimized wavelengths, light reflectors, or diffusing layers can improve light penetration and uniformity (Mitchell et al., 2015). For better mixing and agitation, the optimization of impeller designs, incorporation of airlift pumps, or utilization of paddle-wheel mixers can help maintain culture homogeneity while minimizing shear stress (Kunjapur and Eldridge, 2010). Gas exchange can be improved by integrating spargers, air diffusers, or coiled tubing to facilitate efficient CO_2_ diffusion and oxygen removal (Janssen et al., 2003). Additionally, managing the accumulation of soluble algal products can be addressed through real-time monitoring and automated harvesting systems, which remove excess biomass or regulate nutrient levels (Lim et al., 2022). By incorporating these engineering solutions, the scaling challenges in larger systems can be mitigated, leading to improved productivity and efficiency in large-scale microalgae cultivation.

#### 3.4.2 Effects of 10% harvesting ratio in large scale 10-L *Spirulina* cultivation

**FIGURE 5:**
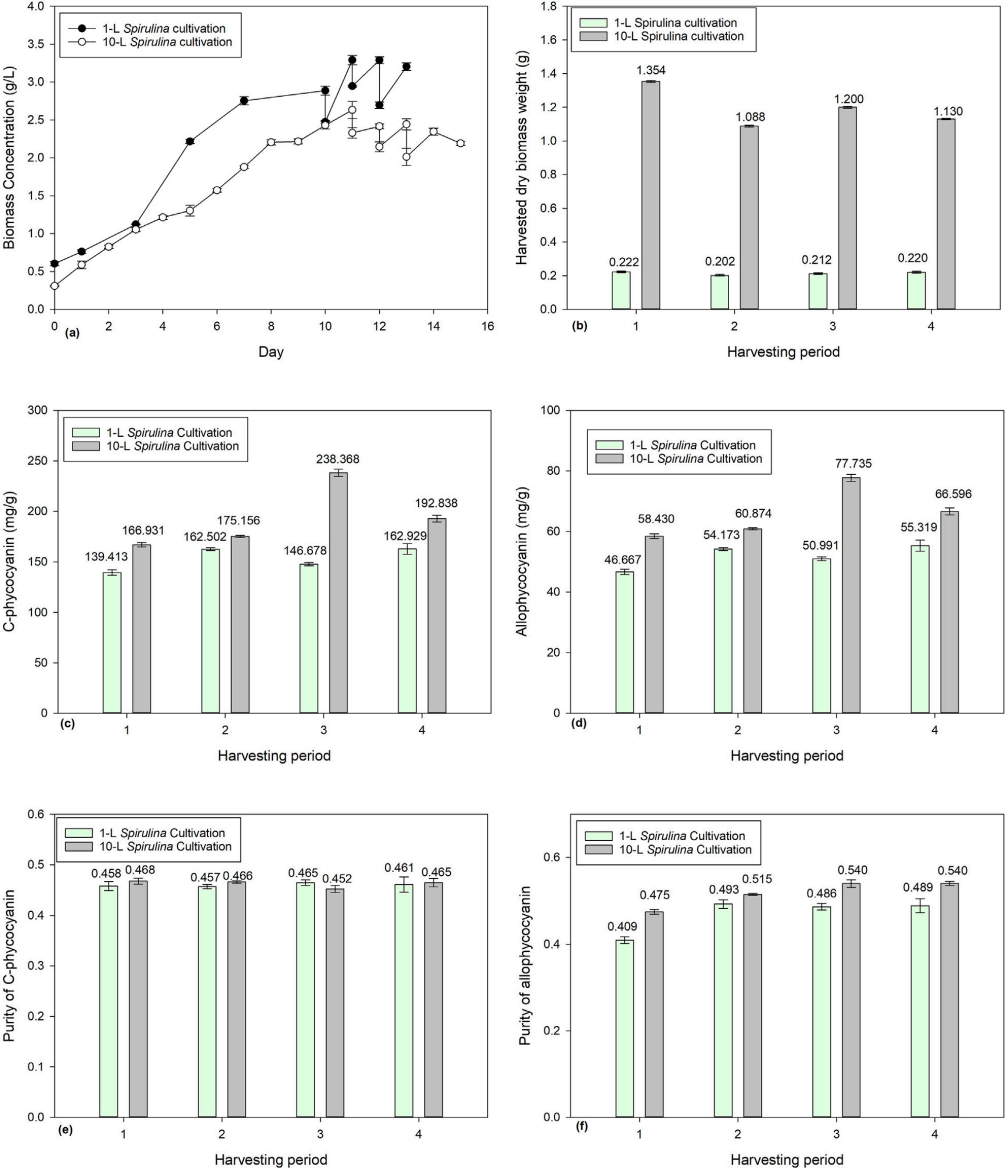
Comparison of 1-L and 10-L *Spirulina* batch culture with 10% harvesting ratio: **(a)** biomass concentration, **(b)** harvested dry *Spirulina* biomass weight, **(c)** C-phycocyanin concentration, **(d)** allo-phycocyanin concentration **(e)** C-phycocyanin purity, and **(f)** allo-phycocyanin purity.

Figure 5a shows the biomass concentration of 1-L and 10-L *Spirulina* batch cultivations. The 1-L batch was cultivated for 10 days while the 10-L batch was cultivated for 11 days before being harvested for 3 consecutive days. Figure 5b demonstrates the results of 10% harvesting from the 10-L batch culture on Days 11, 12, 13, and 14 with a harvested dry biomass weight of 1.354 g, 1.088 g, 1.200 g, and 1.130 g, respectively. On Days 10, 11, 12, and 13, 10% of the 1-L batch culture was harvested with a harvested dry biomass weight of 0.2222 g, 0.2016 g, 0.2120, and 0.2200 g. 10% from 10-L is 1-L in volume and 10% of 1-L is 0.1-L in volume. Thus the results of the 1-L harvested dry biomass weight multiplied by 10 are higher than the harvested dry biomass weight from 10-L culture. This has also shown that the biomass productivity in 1-L batch culture is higher than the 10-L batch culture.

Figures 5c and d shows a comparison of CPC and APC concentration between 1-L and 10-L *Spirulina* batch culture. In the 10-L batch culture, the CPC content range was between 166.9 and 238.4 mg/g and the APC content range between 58.43 and 77.74 mg/g. On the other hand, in the 1-L batch culture, the CPC content ranged between 139.4 and 162.9 mg/g, and APC content ranged between 46.67 and 55.32 mg/g. The 10-L batch culture had higher CPC and APC content than the 1-L batch culture. However, the purity of both CPC and APC had no significant effect over the 4 days of the 10% harvesting ratio.

#### 3.4.3 Effects of NaNO_3_ addition on phycocyanin accumulation in large-scale 10-L *Spirulina* cultivation

**FIGURE 6:**
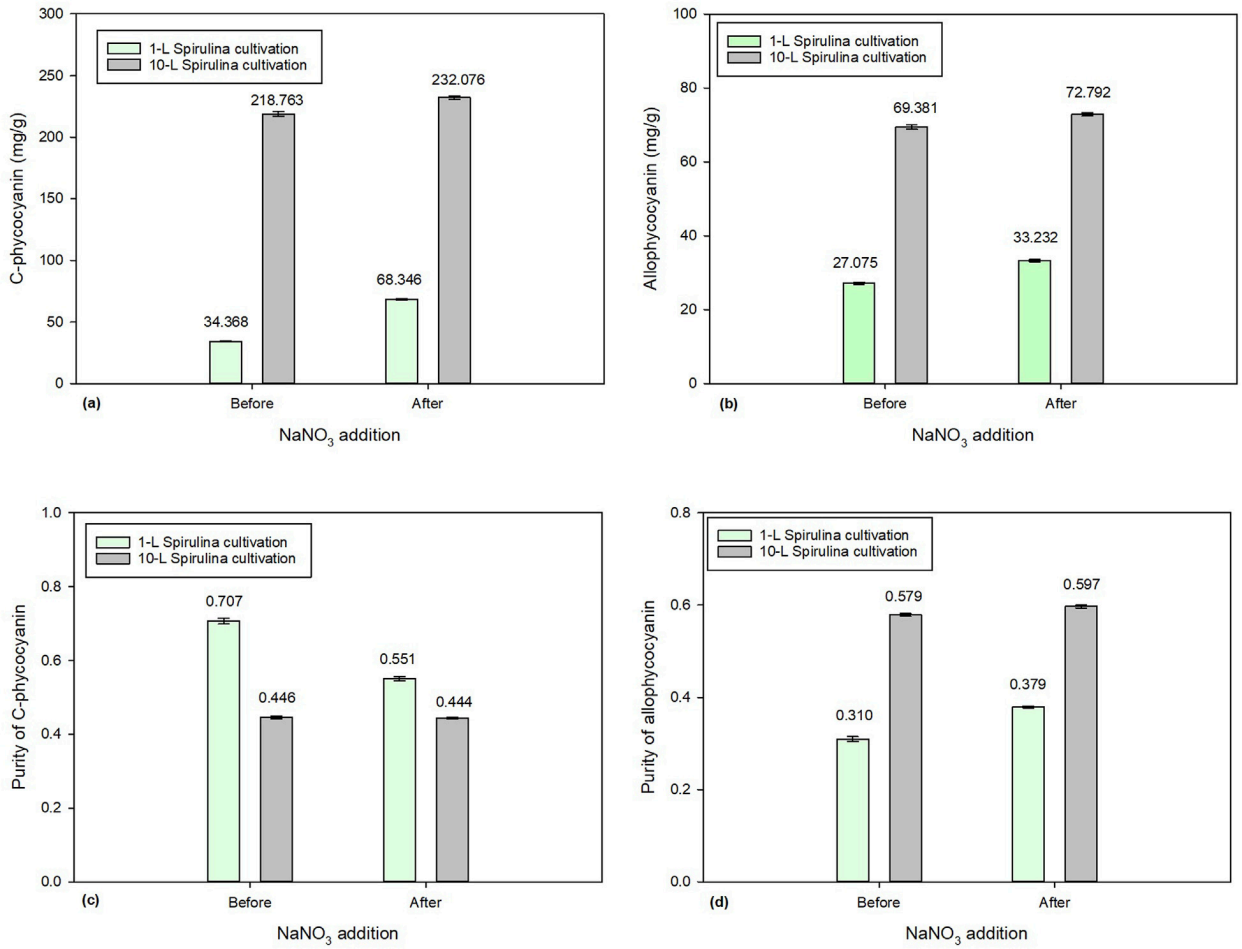
Comparison of 1-L and 10-L *Spirulina* cultivation with the addition of 2.5 g/L of NaNO_3_: **(a)** C-phycocyanin concentration, **(b)** allo-phycocyanin concentration **(c)** C-phycocyanin purity, and **(d)** allo-phycocyanin purity.

Figure 6 compares the effect of 2.5 g/L of NaNO_3_ addition between 1-L and 10-L Spirulina batch cultures. Figures 6a and b show CPC and APC concentrations before and after the addition of 2.5 g/L of NaNO_3_. The initial concentrations of CPC and APC in the 10-L batch culture were 218.76 mg/g and 69.38 mg/g, respectively, which increased to 232.08 mg/g and 72.79 mg/g. Meanwhile, the initial concentrations of CPC and APC in the 1-L batch culture were 34.37 mg/g and 27.07 mg/g, respectively, which significantly increased to 68.35 mg/g and 33.23 mg/g. Both 1-L and 10-L batch cultures showed a significant increase in phycocyanin accumulation due to the addition of 2.5 g/L of NaNO_3_. Surprisingly, the 10-L batch culture had higher phycocyanin accumulation than the 1-L batch.

Although the addition of NaNO_3_ resulted an increase in both CPC and APC concentration and APC purity, it caused the purity of CPC to decrease in both 1-L and 10-L batch cultures (Figure 6c). This would depend on whether the company favored quantity or quality in its end product. The current finding has demonstrated that the addition of NaNO_3_ can enhance the accumulation of phycocyanin in *Spirulina* microalgae, concurrently minimizing chemical usage. However, this approach may not be a sustainable long-term solution since it relies on raw materials extracted from the earth. Further research could explore an alternative nitrogen source that favors an increase in both phycocyanin concentration and purity.

### 3.5 Operating cost analysis

Table 2 shows the operating costs analysis of *Spirulina* cultivation for 1-L and 10-L. The operating cost consists of electricity, water, and chemicals. The cost of electricity is assumed to be the same, using four LED light units of 18 W power each and one air pump unit with a power of 30 W, for both ten 1-L Schott bottles and one 10-L cultivation tank. The cost of water was based on statistics provided by Air Selangor Sdn. Bhd., Malaysia (Air Selangor, 2022). Chemicals costs were based on lab-grade chemical prices (Table 3. The operating costs for both 1-L and 10-L batch cultures was $3.25. The largest expense was electricity at $2.05, followed by chemicals at $1.20. The biomass concentration of *Spirulina* microalgae was higher in the 1-L batch culture, with 2.887 g/L compared to 2.431 g/L in the 10-L batch culture. As a result, the cost of producing 1 kg of *Spirulina* biomass is $ 75.59 in the 1-L batch culture and $ 89.77 in the 10-L batch culture. These results suggest that producing *Spirulina* microalgae in smaller volumes may be cheaper, as it produces a higher yield of dry biomass per liter of culture medium. However, the limitation of this brief calculation has yet to consider the capital cost. The capital cost of ten 1-L vessels could be higher than fabricating a 10-L vessel. Overall, the current findings provide a brief insight into the economic feasibility of producing *Spirulina* biomass in 1-L and 10-L batch cultures. A more detailed economic feasibility of scaling-up *Spirulina* production for commercial scale should be assessed in future studies.

**TABLE 2:** Comparison of operating cost between 1-L and 10-L *Spirulina* microalgae cultivation.

Utilities	1-L	10-L	Justification
Electricity	LED lights: 18 W, 10 days 0.018 kW×24 h×10 days×$ 0.08 per kWh=$ 1.45 Air compressor: 30 W, 10 days 0.030 kW×24 h×10 days×$ 0.08 per kWh=$ 0.60 Total: $1.45 + $0.60 = $2.05		Gao et al. (2016)
Water	0.01 m^3^ × $0.48 per m^3^ = $0.0048		Air Selangor (2022)
Chemicals	See Table 3 for detailed chemicals cost breakdown $0.12 per liter × 10 L = $1.20		Chemical price was source from local supplier
Total Operating Costs	$2.05 (Electricity) + $0.0048 (Water) + $1.20 (Chemicals) = $3.25		
Dry Biomass weight (g/L)	2.887	2.431	
Cost of 1 kg of *Spirulina* Biomass	$75.59	$89.77	

*RM 1.00 = $ 0.21; currency rate based on October 2023.

**TABLE 3:** Detailed Zarrouk chemicals cost breakdown.

Zarrouk	g/L	$/kg	$
NaNO_3_	2.5	6.30	0.01575
K_2_HPO_4_	0.5	12.60	0.00630
MgSO_4_.7H_2_O	0.2	7.35	0.00147
NaCl	1	6.09	0.00609
K_2_SO_4_	1	10.50	0.01050
FeSO_4_.7H_2_O	0.01	7.35	0.00007
NaHCO_3_	18	4.41	0.07938
CaCl_2_.2H_2_O	0.04	8.82	0.00035
Na_2_EDTA	0.08	16.80	0.00134
H_3_BO_3_	0.00286	7.35	0.00002
MnCl_2_.4H_2_O	0.00181	14.70	0.00003
ZnSO_4_.4H_2_O	0.000222	11.34	0.00000
Na_2_MoO_4_	0.0003	37.80	0.00001
CuSO_4_.5H_2_O	0.00007	7.77	0.00000
Co(NO_3_)_2_.6H_2_O	0.00004	77.28	0.00000
Total			$ 0.12 per liter

To improve cost efficiency in larger-scale operations, several cost-reduction strategies could be considered. One approach is integrating renewable energy solutions, such as solar-powered LED lighting combined with battery storage to reduce electricity costs associated with continuous lighting and aeration (Bahadur et al., 2013). Additionally, sourcing nutrients from food waste instead of lab-grade chemicals could significantly lower the cost of culture media while promoting sustainability (Ramandani et al., 2025). Engineering improvements, such as optimizing mixing, aeration, and light distribution in larger systems, could help bridge the productivity gap between small- and large-scale cultivation.

## 4 Conclusion

This study demonstrated a continuous cultivation and harvesting strategy for biomass and phycocyanin production in the biorefinery of *Spirulina* microalgae. The results show that a 10% harvesting ratio provided consistent weights of harvested dry biomass for three consecutive days. Furthermore, NaNO_3_ was found to be the most important nutrient for *Spirulina* cultivation, with the addition of 2.5 g/L significantly improving phycocyanin accumulation. The optimized parameters of a 10% harvesting ratio and 2.5 g/L of NaNO_3_ addition were tested in a 10-L batch culture, which showed decreased biomass productivity compared to the 1-L batch culture, resulting in higher production costs. However, future studies should assess the economic feasibility of scaling up *Spirulina* production for commercial use. Additionally, further research could optimize the concentration of NaNO_3_, NaHCO_3_, and other nutrients for a cost-effective cultivation medium, investigate harvesting frequency, and explore alternative nitrogen sources that increase phycocyanin concentration and purity.

## Data Availability

The raw data supporting the conclusions of this article will be made available by the authors, without undue reservation.
